# Palatable Food Dampens the Long-Term Behavioral and Endocrine Effects of Juvenile Stressor Exposure but May Also Provoke Metabolic Syndrome in Rats

**DOI:** 10.3389/fnbeh.2018.00216

**Published:** 2018-09-19

**Authors:** Eliza Fatima Ali, Jennifer Christine MacKay, Samantha Graitson, Jonathan Stewart James, Christian Cayer, Marie-Claude Audet, Pamela Kent, Alfonso Abizaid, Zul Merali

**Affiliations:** ^1^The Royal’s Institute of Mental Health Research, University of Ottawa, Ottawa, ON, Canada; ^2^Department of Neuroscience, Carleton University, Ottawa, ON, Canada; ^3^School of Psychology, University of Ottawa, Ottawa, ON, Canada; ^4^School of Nutrition Sciences, University of Ottawa, Ottawa, ON, Canada; ^5^Department of Cellular and Molecular Medicine, University of Ottawa, Ottawa, ON, Canada

**Keywords:** juvenile stress, palatable food, metabolic syndrome, dopamine receptors, social interaction, nucleus accumbens, prefrontal cortex, HPA-axis

## Abstract

The juvenile period is marked by a reorganization and growth of important brain regions including structures associating with reward seeking behaviors such as the nucleus accumbens (NA) and prefrontal cortex (PFC). These changes are impacted by stressors during the juvenile period and may lead to a predisposition to stress induced psychopathology and abnormal development of brain reward systems. Like in humans, adult rodents engage certain coping mechanisms such as increases in the consumption of calorie-rich palatable foods to reduce stress, but this behavior can lead to obesity and metabolic disorders. In this study, we examined whether stressors during the juvenile period led to increased caloric intake when a palatable diet was accessible, and whether this diet attenuated adult stress responses. In addition, we examined if the stress buffering effects produced by the palatable diet were also accompanied by an offset propensity towards obesity, and by alterations in mRNA expression of dopamine (DA) receptors in the NA and PFC in adulthood. To this end, juvenile male Wistar rats underwent episodic stressor exposure (forced swim, elevated platform stress and restraint) on postnatal days (PD) 27–29 and received access to regular chow or daily limited access to a palatable diet until adulthood. At the age of 2 months, rats were tested on a social interaction test that screens for anxiety-like behaviors and their endocrine responses to an acute stressor. Animals were sacrificed, and their brains processed to detect differences in DA receptor subtype expression in the PFC and NA using qPCR. Results showed that rats that were stressed during the juvenile period displayed higher social anxiety and a sensitized corticosterone response as adults and these effects were attenuated by access to the palatable diet. Nevertheless, rats that experienced juvenile stress and consumed a palatable diet showed greater adiposity in adulthood. Interestingly, the same group displayed greater mRNA expression of DA receptors at the NA. This suggests that access to a palatable diet mitigates the behavioral and endocrine effects of juvenile stressor exposure in adulthood, but at the cost of metabolic imbalances and a sensitized dopaminergic system.

## Introduction

Childhood and adolescent obesity have doubled in the last two decades where globally 200 million school aged children are now considered overweight or clinically obese (World Obesity, 2014; World Health Organization, 2015). Equally concerning is that some of the diseases previously only seen in adults, have now emerged in the children and youth demographic: overweight and obese children and adolescents being at higher risk for chronic illnesses such as type II diabetes, cardiovascular disease, atherosclerosis and osteoarthritis (Lobstein et al., 2004; Wilkinson and McCargar, 2008; Lee et al., 2014). In terms of prognostic risk, childhood/adolescent obesity can lead to enduring adult obesity (Karnik and Kanekar, 2012).

Although the root cause(s) of increasing prevalence of obesity have not been fully established, the role of stress in obesity has attracted recent attention. In both humans and rodent models, stress increases the intake of palatable food, particularly diets consisting of high carbohydrate, sugar and fat content; the term “comfort food” is often considered to have stress dampening properties of these foods (Dallman, 2010; Yau and Potenza, 2013). In this vein, ingestion of palatable food amid a stressful environment decreases plasma adrenocorticotropic hormone (ACTH) and corticosterone levels, and reduces corticotrophin stimulating hormone (CRH) mRNA expression at the paraventricular nucleus of the hypothalamus, indicating that a palatable diet may dampen hypothalamic-pituitary-adrenal (HPA) axis response to stress (Dallman et al., 2003; Pecoraro et al., 2004; Ulrich-Lai et al., 2007; Christiansen et al., 2011; Zeeni et al., 2012). For instance, access to a high fat cafeteria diet or chocolate cookies was reported to reverse the anxiogenic and depressive traits induced by early-life stressors and normalize CRH expression and basal corticosterone levels in adulthood (Maniam and Morris, 2010a; Marcolin Mde et al., 2012; Krolow et al., 2013; Machado et al., 2013; Kim et al., 2015).

The juvenile period appears to be particularly sensitive to stressors and this may be reflected by enhanced behavioral and endocrine responses to stress when compared to adolescent or adult rodents. For example, juvenile stressor exposure led to greater exploration in the elevated platform maze test as well as higher levels of corticosterone after an acute stressor when compared to stress occurring in adult rats (Jacobson-Pick and Richter-Levin, 2010; Romeo, 2010; Holder and Blaustein, 2014). Furthermore, rats exposed to episodic stressors during the juvenile period showed increased anxiety-like behaviors and these effects were buffered by daily limited access to a palatable diet (MacKay et al., 2014). Access to the palatable food also lowered basal corticosterone levels regardless of juvenile stressor exposure. In this study however, the intake of palatable diet by each of the juvenile-stressed rat was not recorded as the rats were housed in pairs. In contrast to its stress buffering properties, rats that were stressed during the juvenile period and also had access to a palatable diet showed increased adiposity and a trend toward glucose intolerance. This suggested that there is a trade-off where protection against stress during the juvenile period comes at the cost of metabolic imbalance that may predispose individuals to develop obesity and/or metabolic syndrome later in life (MacKay et al., 2014).

Given that the consumption of palatable food attenuates the stress response, some have proposed that palatable food intake is used as a method of self-medication against stress (Dallman et al., 2005; Meye and Adan, 2014). Interestingly, it has been suggested that the mechanisms providing stress relief likely reside at the intersection of reward and stress regulatory circuits (Ulrich-Lai et al., 2010). As palatable diets are highly rewarding and stimulate the release of dopamine (DA), one possible neurochemical mechanism underpinning the stress-reducing effects of palatable foods is through the modification of DA signaling at the mesolimbic “reward” pathway (Berridge, 2009). This pathway includes DA afferents from the ventral tegmental area (VTA) to the nucleus accumbens (NA) and the prefrontal cortex (PFC) which all play a role in stress-induced palatable feeding (Meye and Adan, 2014). Importantly, the PFC and the NA undergo critical re-organization and development during the juvenile period in both humans and rodents (Spear, 2000; Tsoory et al., 2007). Specifically, the expression of DA receptors type 1 and 2 (D_1_ and D_2_ respectively) in the NA and PFC are increased in juveniles (Spear, 2000). It is possible that stressor and/or exposure to palatable diets alter development of these structures leading to adult vulnerability to disease (Maslova et al., 2002; Romeo et al., 2004; Avital and Richter-Levin, 2005; Tsoory and Richter-Levin, 2006; Tsoory et al., 2007; MacKay et al., 2014). For instance, consuming a high fat diet during adolescence resulted in altered dopaminergic activity at the PFC and NA in rodents (Del Rio et al., 2015, 2017; Naneix et al., 2017). In humans, children and adolescents exhibit greater activity in the NA than adults when food rewards were presented, emphasizing that the developing brain is more responsive to reinforcing stimuli (Galvan et al., 2006). Yet, the interplay of palatable diet consumption and stress during the juvenile period and their effects on obesity remains undetermined.

The objectives of this study were to investigate the long-term effects of juvenile stressor exposure and access to a palatable diet on: (1) anxiety-like behaviors, (2) corticosterone response after a mild stressor, (3) feeding and metabolic function and (4) DA receptor mRNA expression at the NA and PFC. We expected that limited access to a palatable diet would ameliorate the effects of juvenile stressor exposure and this would be associated with changes in the DA system.

## Materials and Methods

### Animals

Eighty-eight naïve newly weaned Wistar rats, aged 21-days old, were obtained from Charles River Laboratories (Québec, Canada) and were individually housed in standard plastic rat cages (24.5 cm × 37.5 cm × 19 cm) at a room temperature of 22 ± 1°C on a 12 h light-dark cycle (lights on at 07:00 h and off at 19:00 h). Body weights were measured at their arrival on postnatal day (PD) 21, and every 10 days until PD 70. At PD 60, animals were assigned to one of two testing groups. Group 1 consisted of 40 rats that were used to determine the long-term effects of juvenile stressor exposure and diet on social interaction, adiposity and brain mRNA expression of DA receptors. Group 2 contained 48 rats that were tested for corticosterone levels after a mild stressor and underwent a glucose tolerance test. All procedures were approved by the Animal Care Committee of the University of Ottawa Institute of Mental Health Research and followed the guidelines established by the Canadian Council on Animal Care.

### Study Design

**Figure 1:**
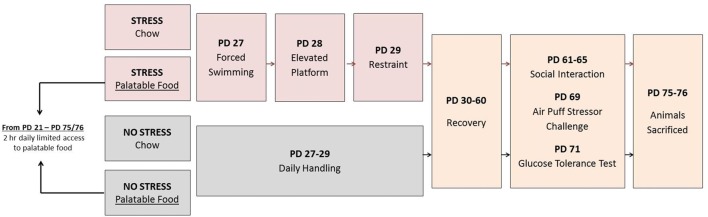
Singly housed animals were assigned to four groups: (1) Chow + Stress; (2) Palatable + Stress; (3) Chow + No Stress and (4) Palatable + No Stress. Groups assigned to the stressor condition were exposed to a different episodic stressor per day from postnatal days (PD) 27–29 while non-stressed animals received daily handling. The following were evaluated in adulthood: Group 1—40 animals (*n* = 10 per group) were tested for behavior (social interaction), adiposity and gene expression. Group 2—48 animals (*n* = 12 per group) were tested for hypothalamic-pituitary-adrenal (HPA) activity after an acute stressor (air-puff) and for glucose tolerance.

In brief, the study design, represented in Figure 1, involved randomly assigning rats to four conditions each with an *n* = 22: Chow + No Stress, Chow + Stress, Palatable + No Stress, Palatable + Stress. Juvenile stressor exposure occurred during PD 27–29 and behavioral, metabolic and endocrine tests, and mRNA expression were analyzed in adulthood. Rats in palatable diet groups were given free access to the diet for 2 h throughout the whole study. The experiments were conducted with rats that were housed individually to track their dietary preferences accurately.

### Diet

All rats were given *ad libitum* access to water and standard laboratory chow (3.4 kcal/g, 4.5% fat, 18.1% protein, 57.3% carbohydrate, Charles River Rodent Diet 5075, Agribrand Purina Canada, Woodstock, ON, Canada). Rats assigned to the palatable diet were given daily limited access (08:00 h to 10:00 h) to a 45% kcal fat diet (4.7 kcal/g, 23.3% fat, 17.3% protein, 47.6% carbohydrates, TD.08811, Harlan Laboratories, Madison, Wisconsin, USA). Access to the palatable food was given on PD 21 and every day until sacrifice (PD 75 or 76). During the juvenile stressor procedure, PDs 27–29, the palatable diet was given prior to stressor exposure. The 24 h chow consumption was measured. Palatable food (2 h) and chow (24 h) consumption was measured every 5 days starting on PD 25 until PD 55. Total caloric intake consisted of the amount consumed from each diet multiplied by energy content (kcal/g). Food preference was a percentage of the palatable food consumed over the total amount of food consumed.

### Juvenile Stressor Procedure

The juvenile stress paradigm was adapted from the 3-day procedure described by Jacobson-Pick and Richter-Levin (2010) and was implemented as described previously (MacKay et al., 2014). In summary, rats were exposed to three consecutive days that comprised of one episodic stressor each on PDs 27–29, starting at 10:00 h. All stressors have been validated to induce corticosterone elevations and the variable schedule was chosen for its consistent robust effects in adult and juvenile rats (Tsoory and Richter-Levin, 2006; Jacobson-Pick and Richter-Levin, 2010).

#### PD 27 Forced Swimming

Rats were individually placed in circular bins filled with water at a depth of 29 cm (bin diameter of 48 cm and height of 42 cm and water temperature 22 ± 2°C) for 10 min after which they were immediately removed from the bin and placed back into their home cages.

#### PD 28 Elevated Platform

Rats were individually placed on a small elevated platform (12 cm × 12 cm; 70 cm elevation from water level) located in a pool filled with water for three sessions of 30 min each. Animals that fell during the stress session were immediately placed back on their platform until 30 min had elapsed. After each session, rats returned to their home cage for 60 min until their next 30 min elevated platform stress session.

#### PD 29 Restraint

Rats were placed in restraining conical plastic bags (30 cm in length) that restricted side to side and forward-backward movement for 30 min. The plastic bag had a small hole at the front end to permit the rats nose to extrude for air ventilation.

### Behavioral Testing—Social Interaction

Starting on PD 61, social interaction was assessed over a period of 3 days, between 10:00–13:00 h daily, under low illumination (30–40 lux). Due to size of study, rats were divided into two testing squads. Rats in the same groups were paired based on weights (less than <10 g difference between pairs), to mitigate stress that could be induced by intimidation as an effect of significant size differences between matched rats (File, 1980; File et al., 2005). On each day, rats were habituated to the testing room for 60 min. On Day 1 (habituation day), paired rats were placed in an open arena (60 cm × 60 cm × 30 cm height of walls) for 5 min to familiarize with each other and reduce the initial social anxiety that might result from having been single-housed throughout the study. Day 2 (habituation day) each rat was individually placed in the arena for 3 min to acclimatize to the environment. Day 3 (test day), the same paired rats were placed in the arena for 7 min and the behaviors of both rats were monitored remotely via a video camera mounted above the arena. Total time spent in social interaction (sniffing, over and under, following, climbing, allogrooming and playfighting) was scored by an examiner blind to the study conditions. Locomotor activity was assessed by counting the number of squares crossed. Rats were immediately returned to their home cages after each habituation or testing session and the open arena was thoroughly cleaned with 70% ethanol between each session.

### Plasma Corticosterone Response to a Mild Stressor

On PD 69, rats were exposed to a mild stressor of an air-puff application. This stressor consisted of a 5 s puff of air from an aerosol can (Emzone, Brampton, ON, Canada) to the rat’s face, delivered once a minute for 5 min. Blood samples were collected, using a tail venipuncture, immediately before the stressor (baseline) and 20, 30, 45, and 75 min post-stressor exposure. Blood droplets were deposited onto 903 Protein Saver filter paper (GE Healthcare Bio-Science Corp, MA, USA), allowed to dry at room temperature and then stored at 20°C.

Blood samples were analyzed using a commercial radioimmunoassay (RIA) as previously described (MacKay et al., 2014). Briefly, 2 days prior to the RIA procedure, blood was eluted from the filter paper by placing one 3 mm punch (per time point) of filter paper in a 12 mm × 75 mm culture tube containing 200 μL Dulbecco’s Phosphate Buffered Saline (Sigma, item D-5773) w/0.1% gelatine, covered with parafilm in a fridge at 4°C. On the day of the RIA procedure, culture tubes containing the samples were placed on an orbital shaker for 1 h at room temperature. Corticosterone levels were determined from the eluted blood sample using commercial RIA kits as per the manufacturer’s instructions (MP Biomedicals, Santa Ana, CA, USA). The inter and intra-assay variability was 11.02% and 9.96%, respectively. The limit of sensitivity of the assay was 5.2 ng/mL.

### Glucose Tolerance Test

On PD 71, following 12 h of fasting, rats were given an injection of 0.75 g/mL dextrose solution (an intraperitoneal dose 1.75 g/kg) as previously described (MacKay et al., 2014). Blood glucose was measured by applying a drop of blood (via tail venipuncture) onto a test strip and then taking a reading with an over the counter glucose measuring sticks and blood glucose meter (Accu-Chek Aviva Nano, Roche Diagnostics, Mannheim, Germany). Glucose levels were assessed immediately before the injection (baseline) and 15, 30, 60 and 120 min post-injection.

### Euthanasia and Brain Tissue Collection

On PD 75 or 76, animals were sacrificed by rapid decapitation and brains were immediately extracted and placed on a stainless steel brain matrix (BSRAS005-1, Zivic) positioned on a block of ice. The matrix had a series of slots spaced approximately 0.5 mm apart that guided razor blades to provide coronal brain sections. Once the brains were sliced, tissue sections from the NA (punched using 18 gauge needle) and PFC (manually dissected) were collected according to the brain atlas of Paxinos and Watson (1998) and placed immediately in nuclease-free tubes positioned on dry ice and stored at −80°C for subsequent determination of the DA receptors, D_1_ and D_2_, mRNA expression. Brain extractions and dissections occurred in an RNase free environment to prevent contamination and RNA degradation. Carcasses were stored at −80°C for later analyses of fat pads.

### Adiposity

Rat carcasses were dissected to obtain visceral, retroperitoneal, subcutaneous and inter-scapular brown fat depots. These depots were weighed on a scale that was accurate at ±0.01 g.

### Real-Time Quantitative Polymerase Chain Reaction (RT-qPCR)

Genes for DA receptors, D_1_ and D_2_, were considered for mRNA expression analysis and are respectively identified as *DRD1* and *DRD2*. Brain sections were homogenized using TriZol^©^ and total RNA was extracted according to the manufacturer’s instructions (Invitrogen, Burlington, ON, Canada). Total RNA concentrations and quality were analyzed using the NanoDrop 2000 spectrophotometer (Thermo-Fisher Scientific). Total RNA was reverse-transcribed using the 5x iScript™ Reverse Transcription Supermix for Real-Time quantitative Polymerase Chain Reaction (RT-qPCR) and a T100 Thermal Cycler (Bio Rad Laboratories). Afterwards, aliquots of cDNA were analyzed, in duplicates with Sso Advanced™ Universal SYBR^©^ Green Supermix and a CFX96 Touch Real-Time PCR Detection System (Bio-Rad Laboratories).

Primers that amplify β-actin (*Actβ*) and ribosomal protein L-19 (*RPL19*) were used as reference genes and utilized for gene expression normalization such that the averaged cycle quantification value (C_Q_) of the two reference genes was subtracted from the C_Q_ of each gene of interest within the PFC and NA (ΔC_Q_). Calculation of the mRNA fold changes was conducted using the 2^−ΔΔ^CQ method (Livak and Schmittgen, 2001) which converted ΔC_Q_ values relative to Chow + No Stress group (calibrator). Analysis of samples and results met the minimum requirements for publication of RT-qPCR experiments (MIQE) guidelines. Primer efficiency and the cycle threshold were determined from the slope in relation to the absolute copy of RNA quantity and the C_Q_ values using Bio-Rad Amplification CFX Manager™ Software version 3.0. All primer pairs had a minimum efficiency of 90%.

Primer sequences selected for RT-qPCR analysis as follows:

*Actβ* forward: 5′TAT GCC AAC ACA GTG CTG TCT GG 3′
*Actβ* reverse: 5′TAC TCC TGC TTC CTG ATC CAC AT 3′
*RPL19* forward: 5′TGC AGC CAT GAG TAT GCT TAG 3′
*RPL19* reverse: 5′GAG AGT TGG CAT TGG CGA TT 3′
*DRD1* forward: 5′GGT CCA AGG TGA CCA ACT TCT 3′
*DRD1* reverse: 5′CCC AGA TGT TAC AAA AGG GAC C 3′
*DRD2* forward: 5′AAG ACA CCA CTC AAG GGC AAC 3′
*DRD2* reverse: 5′ATC CAT TCT CCG CCT GTT CAC 3′

### Statistical Analyses

All statistical analyses were conducted using IBM’s Statistical Package for Social Sciences (IBM SPSS statistics version 23). Caloric intake, chow consumption (g) and body weight across age were analyzed using repeated measures analysis of variance (ANOVA) with Diet (Chow vs. Palatable) and Stress (No Stress vs. Stress) as the between group variables and Age as the within group variable. Palatable food preference (%) and consumption (g) was analyzed using repeated measures ANOVA with Stress as the between-group variable and Age as the within-group variable. Caloric intake relative to weight was determined for PD 30 (after juvenile stressor exposure) and PD 55 (late adolescence approaching adulthood) using two-way ANOVA. Body weight gained from PD 27 to PD 30 and area under the curve (AUC) for glucose concentrations (for the glucose tolerance test) were analyzed using non-parametric tests as data was not normal. Body weight gained from PD 21 to PD 70 and weight of adipose tissue was analyzed using two-way ANOVA with Diet and Stress as the between-group variables. Corticosterone response to a mild stressor and glucose tolerance results were analyzed using repeated measures ANOVAs, with Diet and Stress as the between-group variables and Time as the within group variable. In the case of significant interaction effects, *post hoc* comparisons using *t*-tests with Bonferroni correction were conducted. The critical value for significance was set at *α* = 0.05. For repeated measures analysis, unless otherwise stated, the *F* values specified represents when the assumption of sphericity was met. Data points ±3 standard deviations from calculated means were considered outliers and removed from the statistical analysis, hence the N and df associated with these measures vary across outcomes (Taylor, 1997). In the social interaction test, glucose tolerance test and the mild stressor test, there was one outlier that was removed from the data sets. In addition, a number of samples were excluded from qPCR analyses due to technical difficulties (contaminated or missing samples). Graphical analysis, Levene’s and Shapiro-Wilk tests were used to check for model assumptions (normality and constancy of variance). Values represented are means ± standard error mean (SEM).

## Results

### Juvenile Stressor Exposure Decreased Caloric Intake Only in Chow-Fed Rats

**Figure 2:**
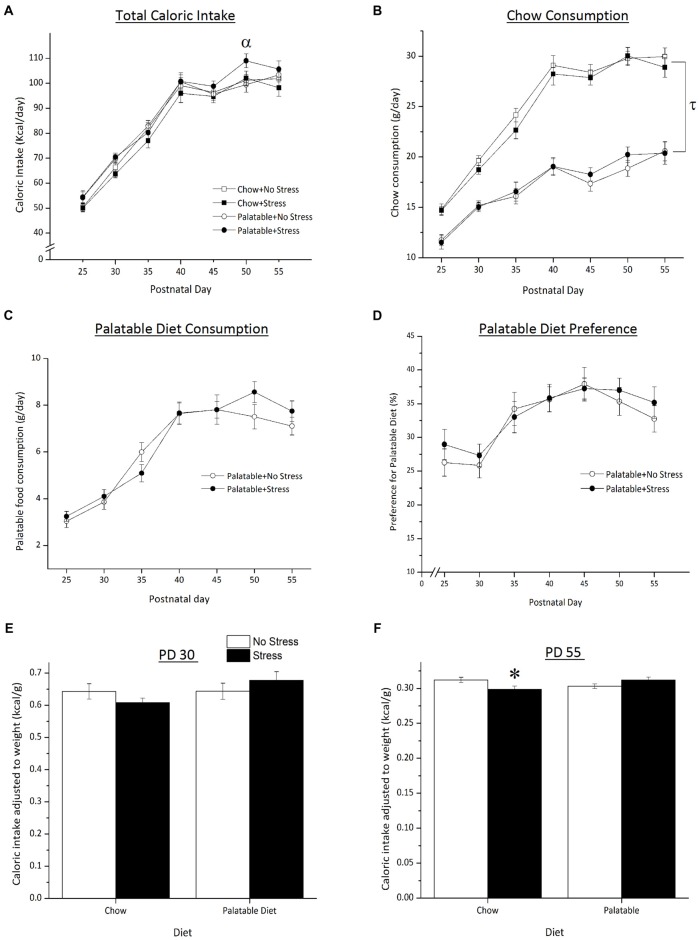
Effect of access to a palatable diet and juvenile stressor exposure on total caloric intake, chow and palatable diet consumption and caloric intake (adjusted to body weight), palatable diet preference and weight gained. **(A)** Access to a palatable diet and juvenile stressor exposure increased total caloric intake on PD 50. **(B)** Access to a palatable diet led to an overall lower chow consumption across age. **(C)** Palatable diet consumption did not differ regardless of stressor exposure. **(D)** Across development, juvenile stressor exposure did not elicit a preference for a palatable diet. **(E)** Caloric intake did not differ on PD 30 between groups right after 3-day juvenile stressor exposure (PD 27–29). **(F)** Juvenile stressor exposure decreased caloric intake in late adolescence (PD 55) and this did not occur in rats that consumed the palatable diet. ^α^Significantly different from non-stressed palatable diet fed group (*p* < 0.05). ^τ^Significantly different to chow diet groups (*p* < 0.05). *Significantly different from stressed palatable diet group and non-stressed chow group (*p* < 0.05).

As shown in Figure 2, total caloric intake varied as a function of the Age × Stress interaction, *F*_(6,504)_ = 2.85, *p* < 0.05 (Figure 2A). Simple effects indicated that on PD 50, the caloric intake was greater in palatable food fed rats that experienced the juvenile stress when compared to non-stressed rats consuming the same diet (*p* < 0.05).

Access to a palatable diet resulted in a decrease in chow intake regardless of animals being stressed or not. Statistical analyses showed that total chow intake was significantly lower in palatable diet fed rats when compared to rats that consumed chow only (Figure 2B, main effect of Diet, *F*_(1,83)_ = 148.43, *p* < 0.05). There was no main effect of Stress or a Diet × Stress interaction (*p* > 0.05). There were no significant differences in palatable diet consumption (Figure 2C) between stressed and non-stressed rats across age (no Stress × Age interaction, *F*_(6,252)_ = 4.08, *p* > 0.05). Preference for the palatable food did not differ between non-stressed and stressed rats (no main effect of Stress *F*_(1,42)_ = 0.18, *p* > 0.05; Figure 2D).

Caloric intake adjusted to body weight (caloric intake per gram of weight gained) is represented in Figures 2E,F. On PD 30, after the last day of juvenile stressor exposure, the relative caloric intake did not differ across groups (all effects *p* > 0.05). However, on PD 55, there was a significant Stress × Diet interaction (*F*_(1,83)_ = 7.37, *p* < 0.05) where rats that were exposed to juvenile stress and had access to the chow only consumed less calories than to chow-fed non-stressed rats (*p* < 0.05) and stressed rats fed the palatable diet (*p* < 0.05).

### Palatable Diet Led to Greater Weight Gain, but Stressed Rats Showed Increased Adiposity in Adulthood

**Figure 3:**
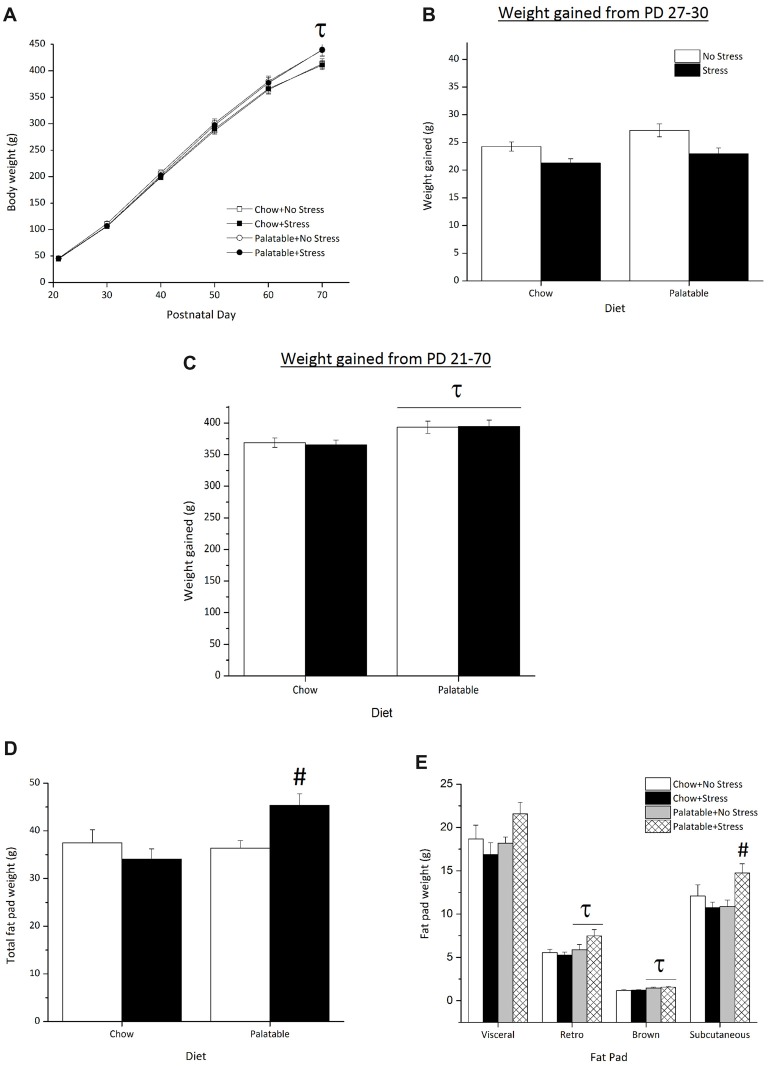
Effect of access to a palatable diet and juvenile stressor exposure on body weight and adiposity.** (A)** Consumption of a palatable diet led to greater body weight on PD 70.** (B)** There were no significant differences in weight gained from PD 27–30 shortly after juvenile stressor exposure **(C)** Access to the palatable diet increased overall weight gained from PD 21–70. **(D)** Access to a palatable diet increased total adiposity in juvenile stressed rats only. **(E)** Access to a palatable diet and juvenile stressor exposure increased subcutaneous fat, whereas access to a palatable diet increased retroperitoneal and brown fat. ^τ^Significantly different to chow diet groups (*p* < 0.05). ^#^Significantly different from other conditions (*p* < 0.05).

Body weights across age are represented in Figure 3A. There was an Age × Diet interaction (*F*_(5,415)_ = 7.04, *p* < 0.05) and simple effects comprising this interaction found that on PD 70, rats that had access to the palatable diet, regardless of stressor exposure, weighed significantly more than their chow-fed counterparts. The main effect for Stress or the interaction between Diet and Stress were not significant (*p* > 0.05).

Figure 3B represents the weight gained from PD 27 (weighed prior to stressor exposure) to PD 30 (a day after the last stressor procedure). A Kruskal Wallis test showed no statistically significant difference in weight gained across groups after juvenile stressor exposure ($\chi_{\left(3\right)}^{2}$ = 7.5, *p* > 0.05). As seen in Figure 3C, rats that consumed the palatable diet gained the most weight by adulthood (PD 70), irrespective of stressor exposure, when compared to chow-fed rats only (main effect of Diet, *F*_(1,83)_ = 9.38, *p* < 0.05).

Figure 3D shows the average total fat pad weight across all experimental groups. Total fat pad weight was significantly higher in rats that had access to the palatable diet (main effect of Diet, *F*_(1,35)_ = 5.02, *p* < 0.05). A significant Stress × Diet interaction effect (*F*_(1,35)_ = 7.42, *p* < 0.05) followed by *post hocs* determined that rats that were stressed during the juvenile period and consumed the palatable diet accumulated more overall fat than all other groups (*p* < 0.05). Figure 3E shows the weights of the individual fat pads. Rats that had access to the palatable diet and were stressed during the juvenile period seemed to have accumulated more visceral fat compared to rats in other conditions, although this difference failed to attain statistical significance (Stress × Diet interaction, *F*_(1,35)_ = 7.42, *p* = 0.057). In addition, access to the palatable diet led to larger retroperitoneal fat pads (main effect of Diet (*F*_(1,35)_ = 5.66, *p* < 0.05) and brown fat pads (main effect of Diet, *F*_(1,35)_ = 13.75, *p* < 0.05) regardless of stressor exposure. There was a significant Stress × Diet interaction (*F*_(1,35)_ = 6.99, *p* < 0.05) for subcutaneous fat pad weight, indicating that rats stressed during the juvenile period that consumed the palatable diet had more subcutaneous fat compared to all other groups (*p* < 0.05).

### Stress During the Juvenile Period Resulted in Higher Corticosterone Levels at Baseline and Following Exposure to an Acute Stressor in Adulthood, and the HPA Response Was Attenuated by Access to a Palatable Diet

**Figure 4:**
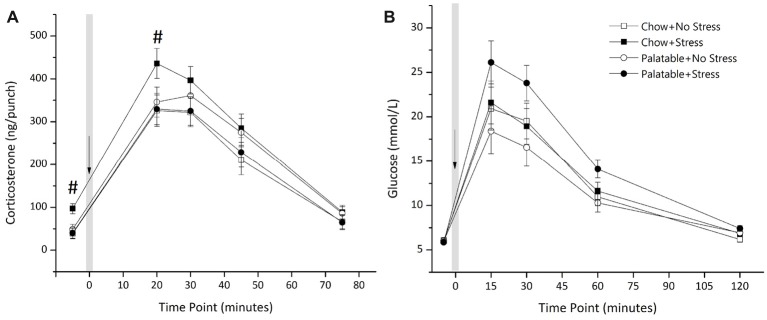
Effects of access to a palatable diet and juvenile stressor exposure on glucose tolerance and HPA-axis response to a mild stressor. **(A)** Juvenile stressor exposure increased corticosterone levels at baseline and 20 min after an air-puff stressor in adult chow-fed rats but not in rats that had access to the palatable diet. Air-puff administered at *t* = 0 as indicated and the volume of each punch was 2.5 μL. **(B)** Glucose levels were elevated at 15, 30 and 60 min after intraperitoneal administration of dextrose (0.75 g/mL) at *t* = 0 min, as indicated, in all groups. However, there was no significant effect of diet or stress conditions. ^#^Significantly different from other conditions (*p* < 0.05).

Figure 4A shows plasma corticosterone after exposure to a mild stressor (puffs of air). There was a significant interaction of Stress × Diet (*F*_(1,42)_ = 4.18, *p* < 0.05) with no main of effect of Diet or Stress (*p* > 0.05). Simple effects analysis showed that rats that experienced juvenile stressor exposure and had access to chow only led to elevated corticosterone levels at baseline and at 20 min post air-puff administration (*p* < 0.05) and this elevation of corticosterone levels was not observed in rats that consumed the palatable diet and experienced juvenile stress.

Figure 4B shows glucose concentration in animals from the four experimental groups after an administration of dextrose. There was no main effect of Diet (*F*_(1,43)_ = 0.387, *p* > 0.05) or a Stress × Diet interaction (*F*_(1,43)_ = 2.76, *p* > 0.05). The main effect of Stress was not significant (*F*_(1,43)_ = 3.64, *p* = 0.063). A Kruskal Wallis test showed no statistically significant difference in AUC for glucose concentration across groups ($\chi_{\left(3\right)}^{2}$ = 6.63, *p* > 0.05). Mean (±SEM) AUC for glucose concentrations of each group was as follows: Chow + No Stress 115.11 ± 12.22; Chow + Stress 117.17 ± 10.86; Palatable + No Stress 100.32 ± 10.72; Palatable + Stress 141.29 ± 6.90.

### Palatable Diet Attenuated Anxiety-Like Behaviors Induced by Juvenile Stressor Exposure in the Social Interaction Test

**Figure 5:**
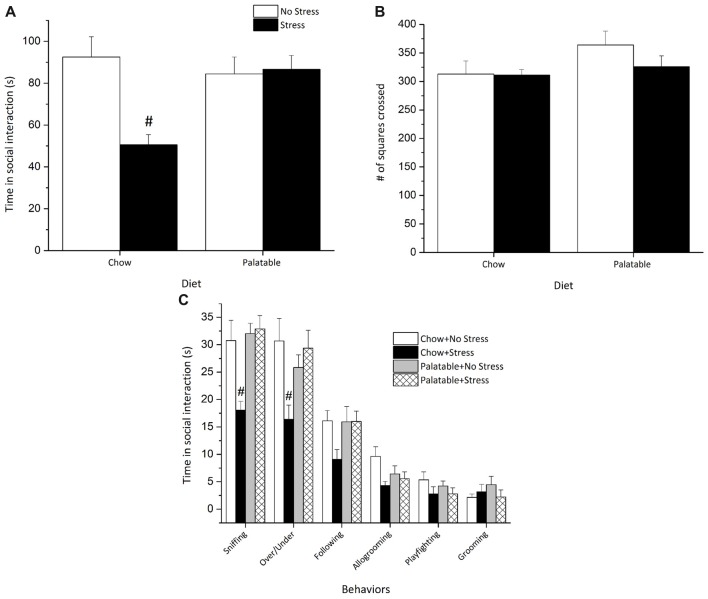
Access to a palatable diet prevents the decrease in social interaction in adulthood elicited by juvenile stressor exposure. **(A)** Adult rats exposed to juvenile stress exhibited an overall reduction in total social interaction and this effect was reversed in palatable diet fed rats. **(B)** Locomotor activity (# of squares crossed) was comparable across groups. **(C)** Juvenile stressor exposure decreased sniffing and over/under behaviors. ^#^Significantly different from other conditions (*p* < 0.05).

The social interaction test was used to examine behavioral responses associated with anxiety-like behaviors in rats including specific patterns of social interaction and locomotor activity. Social interaction measures were used to assess whether access to palatable food would modulate the long-term behavioral effects of juvenile stressors exposure. A significant Stress × Diet interaction (*F*_(1,35)_ = 9.53, *p* < 0.05) followed by *post hoc* tests revealed that rats exposed to juvenile stress and only given chow spent less time in social interaction than all other groups (*p* < 0.05) and this decrease in social interaction was not observed in stressed rats with access to a palatable diet (Figure 5A).

None of the experimental manipulations affected locomotor activity as measured by line crossings in the open field box (*p* > 0.05), suggesting that locomotor activity was not altered by any of the manipulations (Figure 5B).

Figure 5C represents a breakdown of the individual social behaviors. Sniffing and over/under behaviors varied significantly as revealed by two-way ANOVAs. Significant Stress × Diet interaction effects followed by *post hoc* comparisons revealed that rats exposed to juvenile stress and given access to chow only sniffed less (*F*_(1,35)_ = 7.54, *p* < 0.05) and displayed fewer over/under behaviors (*F*_(1,35)_ = 8.32, *p* < 0.05) than all other groups. Allogrooming was effected by Stress (*F*_(1,35)_ = 5.77, *p* < 0.05), but there was no significant Diet effect or a Stress × Diet interaction (*p* > 0.05), suggesting that allogrooming was decreased by stress but not influenced by palatable diet exposure. Finally, no significant differences were recorded for other behaviors like following, playfighting and grooming (*p* > 0.05).

### DA Receptor Expression in the PFC and NA

**Figure 6:**
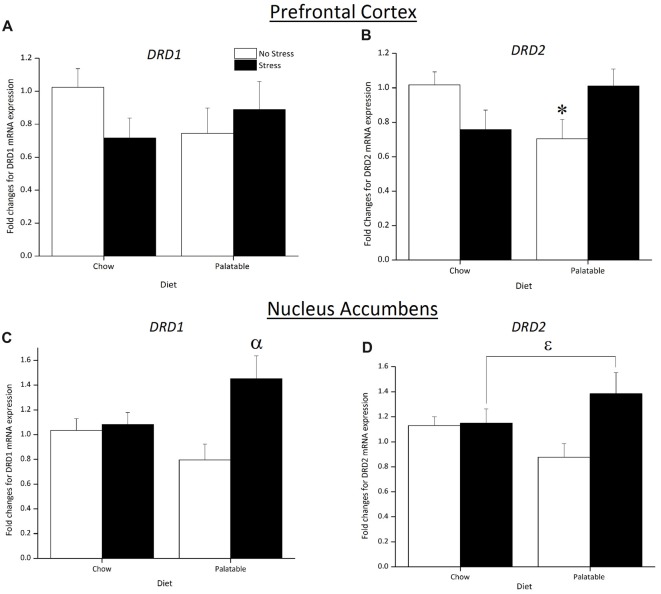
Effect of juvenile stress and diet on fold changes for dopamine (DA) receptors mRNA expression in the prefrontal cortex (PFC) and nucleus accumbens (NA). **(A)** There were no significant changes in *DRD1* expression across groups in the PFC. **(B)** *DRD2* expression in the PFC was downregulated in non-stressed rats fed the palatable diet. **(C)** There was an upregulation in *DRD1* expression in juvenile stressed rats with access to palatable food in the NA **(D)** *DRD2* expression was upregulated in juvenile stressed rats with access to palatable food however this did not reach significance in the NA. *Significantly different from stressed palatable diet group and non-stressed chow group (*p* < 0.05). ^α^Significantly different from non-stressed palatable diet group (*p* < 0.05). ^ε^Significantly different from non-stressed groups (*p* < 0.05).

Figure 6 shows changes in mRNA expression for the DA receptors in the PFC and NA. In the PFC, *DRD1* expression was not affected by Stress, Diet or the interaction of Stress × Diet (*p* > 0.05; Figure 6A). In contrast, a significant Stress × Diet interaction (*F*_(1,31)_ = 7.37, *p* < 0.05) followed by *post hoc* analysis revealed that *DRD2* expression was downregulated in non-stressed rats that consumed the palatable diet only and this was not observed in chow-fed rats (*p* < 0.05) or previously stressed rats that consumed the same diet (*p* < 0.05; Figure 6B).

A significant main effect of Stress (*F*_(1,21)_ = 6.70, *p* < 0.05) and Stress × Diet interaction (*F*_(1,21)_ = 4.99, *p* < 0.05) indicated that *DRD1* expression in the NA was upregulated in rats that were stressed and consumed a palatable diet compared to non-stressed palatable diet fed rats (*p* < 0.05; Figure 6C). As observed in Figure 6D, an overall significant main effect of Stress (*F*_(1,21)_ = 4.22, *p* < 0.05) determined that *DRD2* expression was upregulated.

### Discussion

Stressful experiences during the juvenile period or adolescence and the consumption of high calorie “comfort food” contribute to childhood and the onset of adult obesity. This supports the notion that children will consume palatable foods as a method of coping with stress when other coping skills have not fully developed (Michels et al., 2013, 2015a,b). In the current study, using our model of juvenile stress, we showed that daily limited access to a palatable diet mitigated the behavioral and endocrine effects in rats exposed to stress during juvenility. However, access to this diet had detrimental metabolic consequences and altered gene expression in areas involved in reward in adulthood. These changes resulted in excess weight and could ultimately lead to obesity and metabolic syndrome.

One of the main highlights of our study was a reversal of social deficits in juvenile stressed rats that consumed a palatable diet. The reduced social behaviors in a novel environment can be attributed to increased anxiety-like traits (File, 1980; File et al., 2005), which were only present in juvenile stressed rats that consumed chow. However, access to a palatable diet during the juvenile period and throughout the lifespan completely mitigated the long-term anxiogenic effects of juvenile stress. The current results provide further evidence that access to a palatable diet can protect against the long-term social consequences of juvenile stressor exposure. Similar stress-buffering characteristics of a high fat diet (*ad lib* access) have been observed in adult rats exposed to prolonged maternal separation. In these studies, the high-fat diet normalized anxiety-like behavior in the elevated plus maze, open field test and light-dark box test, in maternally separated rats when tested in late adulthood (Maniam and Morris, 2010a, b; Marcolin Mde et al., 2012; Kim et al., 2015). Though in our study the stressor was administered during juvenility and access to the palatable diet was limited, our results further support the possibility that a high fat diet can mitigate the persisting effects of stressor exposure during key developmental periods.

Interestingly, our data show that, regardless of the diet, stressed animals consumed more calories overall compared to non-stressed controls. Nevertheless, when we examined caloric intake in proportion to body weight, this difference disappeared. In fact, by PD 55, caloric intake in proportion to body weight was lower in stressed chow-fed rats. This might be because rats that consumed the palatable diet gained more weight than those that had access to the chow diet only, regardless of stressor exposure. But in spite of caloric intake in proportion to body weight not being higher in stressed rats, juvenile stressor exposure in combination with access to a palatable diet resulted in greater total adiposity. Moreover, the effect of stress on adiposity was seen in several fat pads, but greater accumulation of fat was seen in subcutaneous fat pads from rats that were stressed and that had access to the palatable diet. We previously demonstrated in pair-housed rats that access to a palatable diet increased adiposity regardless of juvenile stressor exposure (MacKay et al., 2014). In this present experiment, it is possible that the chronic social isolation during development combined with the juvenile stressor exposure led to this adipose accumulation when a palatable diet was provided. Supporting this contention, *ad lib* access to a high fat diet and social isolation between PD 21–28 exacerbated abdominal adiposity (Arcego et al., 2014). Furthermore, increased glucocorticoid levels due to chronic stress have been associated with increased fat storage (Dallman et al., 2005) and similar to our study, access to a high fat diet prior to stressor exposure increased adiposity afterwards (Kamara et al., 1998).

Along with increased weight and adiposity, another indicator of metabolic syndrome is altered glucose metabolism. There is strong evidence that consumption of a high fat diet can lead to dysfunction in glucose metabolism (Paternain et al., 2011; Mayans, 2015; Dalby et al., 2017; Wu et al., 2018). In this study, we did not see an effect of the palatable diet or stress on glucose tolerance replicating previous data from our lab (MacKay et al., 2014). Many studies have indicated that blood glucose levels become elevated following acute (Tannenbaum et al., 1997; Ghalami et al., 2013) or chronic stress (García-Díaz et al., 2007; Devaki et al., 2013), and these elevated levels were observed shortly after or during stressor exposure. The increase in glucose is a mechanism to mobilize energy to skeletal muscles to cope with the immediate stressor at hand (Sapolsky et al., 2000). It is therefore likely that these juvenile stressors are not sufficient to alter glycemic responses during adulthood.

Baseline and stressor induced corticosterone levels were significantly elevated in our chow-fed stressed rats in adulthood and not in those fed the palatable diet. Access to a palatable diet reduced basal corticosterone concentrations and corticosterone responses to an acute stressor to be comparable to the non-stressed rats. This supports previous work showing that access to cookies prevents the effects of early life stress on basal corticosterone levels in adolescent rats (Lee et al., 2014). Similarly, elevated levels of fecal corticosterone were found in chronically stressed rats and not in those that consumed a cafeteria diet (Paternain et al., 2011). Access to a palatable diet has also been associated with reduced hypothalamic CRF mRNA (Foster et al., 2009; Ulrich-Lai et al., 2010) and reduced levels of corticosterone and ACTH in response to restraint stress in adult rats (Pecoraro et al., 2004; Ulrich-Lai et al., 2010). Our results show that the palatable diet attenuated the effects of juvenile stress exposure on corticosterone secretion and normalized corticosterone levels in response to a mild stressor later in life.

The HPA-axis can directly influence the mesolimbic dopaminergic pathway. Acutely, stress can trigger the release of CRH into the VTA to stimulate DA into the NA, an event that is associated with increased reward seeking behaviors that help mitigate the negative effects produced by the stressor (Dallman et al., 2003, 2005). Repeated stressor exposure, however, can lead to alterations in this system that produce behavioral deficits linked to depression (Patterson and Abizaid, 2013). As such, the reward pathway contributes to the stress-buffering effects of palatable food, but chronic exposure to stress alters this pathway. Here we examined whether access to palatable food could alter the long-term impact of juvenile stressor exposure on mRNA expression of DA receptors in the PFC and NA in stressed and non-stressed rats. These structures were selected as they play important roles in cognitive processes and reward respectively and are particularly sensitive to stress during juvenile and adolescent development (Spear, 2000; Andersen, 2003; Burke and Klaus, 2014). In addition, DA receptors, such as D_1_ and D_2_ play an important in modulating the response to a rewarding stimulus, like a high fat diet (Stice et al., 2011; Volkow et al., 2013). Our results show that juvenile stress resulted in higher D_1_ and D_2_ receptor mRNA expression (*DRD1* and* DRD2*, respectively) in the NA of stressed rats that had access to a palatable food. In contrast, consumption of a high fat and high sugar diets have been shown to decrease D_1_ and D_2_ receptor protein and mRNA expression (Alsiö et al., 2009, 2010; Johnson and Kenny, 2010; Ong et al., 2013; Kovalenko et al., 2014). Importantly, it seems that stress can increase the expression of D_1_ and D_2_ receptors in the NA as found by others (Hill et al., 2014; Lakehayli et al., 2015; Said et al., 2015) and that this effect could be enhanced by access to a high palatable diet. The mechanisms for this effect require further investigation but it is conceivable that juvenile stressor exposure could cross-sensitize with food reward in rats fed the palatable diet. Indeed, palatable food activates similar brain reward circuits to that of drugs of abuse (Volkow and Wise, 2005; Volkow et al., 2013). There is evidence showing that stressor exposure during adolescence produced a sensitization for drugs of abuse such as potentiated locomotor activity to acute amphetamine (Peleg-Raibstein and Feldon, 2011; Burke et al., 2013; Burke and Klaus, 2014) and cocaine (Lepsch et al., 2005) administration in adulthood.

In addition to changes in *DRD1* and *DRD2* expression in the NA, we also found that non-stressed rats with access to a palatable also showed a decrease in *DRD2* expression in the PFC, but this effect was not seen in stressed rats with access to the same diet. Carlin et al. (2013) made similar observations in mice that were fed a high fat diet from weaning and until these animals were 4 months old. The downregulation of *DRD2* expression linked to the palatable diet was not apparent in juvenile stress exposed rats. Other mechanisms triggered by juvenile stress could be at play to prevent the downregulation of *DRD2* expression by a high fat diet. D_2_ is an autoreceptor, modulating DA synthesis during adolescence and then synaptic release solely in adulthood (Andersen et al., 1997). It has been shown that repeated social stress during early adolescence caused DA overflow in the PFC activating D_2_ receptors to reduce DA synthesis (Burke et al., 2013). It is possible that this temporary reduction in DA content in the PFC due to pre-pubertal stress could drive the mRNA expression of *DRD2* to normal levels later in adolescence to maintain normalized DA release in adulthood. This could override undefined mechanisms involved in high fat diet induced downregulation of this receptor seen in non-stressed palatable fed rats.

In general, the results from our study were complex and difficult to evaluate given the multitude of variables examined. Given this complexity, it is not surprising that behavioral, endocrine and molecular measures do not seem to correlate well with each other. This might be in part given the number of manipulations conducted as part of the study as well as those that were required to be able to measure individual caloric intake like isolation. Indeed, the purpose of housing rats individually was to provide a more accurate measure of palatable food preference. This, however, is also a stressor (Fone and Porkess, 2008) and likely impacted non-stressed chow-fed animals and might have been the reason why we did not observe changes in caloric intake following stress that have been reported by other studies (Pecoraro et al., 2004; Dallman et al., 2005; Dallman, 2010; Toledo-Rodriguez and Sandi, 2011; Thompson et al., 2015). Moreover, the time of palatable diet access may have also been a factor. In our study we restricted access to the palatable diet to 2 h in the early light phase to determine if the rats would binge on the diet during this time. Perhaps a stronger preference for this diet would have been observed if the diet was provided after the stress or during the night time period as evident in other studies investigating adolescent stress and diet (Toledo-Rodriguez and Sandi, 2011; Handy et al., 2016). Nevertheless, our results do reflect the additive effects that additional stressors can produce on top of the isolation and how these can be affected by diet.

In conclusion, our results demonstrated that access to a palatable diet can mitigate the effects of juvenile stressor exposure into adulthood. However, this occurred at the cost of detrimental alterations in metabolism, such as increased adiposity. Our findings suggest that a palatable diet can provide stress-buffering effects, ameliorating anxiety-like characteristics and normalizing basal corticosterone levels. This also led to altered DA receptor mRNA expression in areas involved in reward. Our evidence provides strength to the idea that ingestion of comfort food during juvenility can facilitate coping with stress, however, the long-term effects of this can lead to the devastating metabolic consequences of obesity.

## Author Contributions

JM, PK, AA and ZM conceived and designed the experiments. EA, CC, JM, JJ and SG performed the experiments. EA, CC, JM, M-CA and PK analyzed the data. EA, JM and PK wrote the article. AA, M-CA, PK and ZM manuscript revision.

## Conflict of Interest Statement

The authors declare that the research was conducted in the absence of any commercial or financial relationships that could be construed as a potential conflict of interest.
